# The co-existence of elevated high sensitivity C-reactive protein and homocysteine levels is associated with increased risk of metabolic syndrome: A 6-year follow-up study

**DOI:** 10.1371/journal.pone.0206157

**Published:** 2018-10-23

**Authors:** Jinkwan Kim, Sangshin Pyo, Dae Wui Yoon, Seungkwan Lee, Ja-Yun Lim, June seok Heo, Seungku Lee, Chol Shin

**Affiliations:** 1 Department of Biomedical Laboratory Science, College of Health Science, Jungwon University, Geo-San, Republic of Korea; 2 Department of Integrated Biomedical and Life Sciences, College of Health Science, Korea University, Seoul, Korea; 3 Institute of Human Genomic Study, Korea University Ansan Hospital, Korea University, Ansan, Republic of Korea; 4 Division of Pulmonary, Sleep, and Critical Care Medicine, Department of Internal Medicine, Korea University Ansan Hospital, Ansan, Republic of Korea; Mathematical Institute, HUNGARY

## Abstract

Accumulating evidence has revealed that both high sensitivity C-reactive protein (hsCRP) and homocysteine (HCY) are associated with increased risk of metabolic syndrome (MetS) and cardiovascular disease. However, it is unclear whether the coexistence of these conditions accelerates the risk of metabolic syndrome (MetS). We hypothesized that the combination of high sensitivity C-reactive protein (hsCRP) and homocysteine (HCY) levels could exacerbate the development of MetS in a large prospective cohort study. We selected data from 3,170 individuals (1,614 men and 1,556 women) who participated in the Korean Genome and Epidemiology Study. Participants with high hsCRP and HCY levels were categorized into quartiles. MetS was defined based on the criteria of the modified National Cholesterol Education Program, Adult Treatment Panel III. The prevalence of MetS was higher in participants with concurrent high hsCRP and HCY compared to those with low hsCRP and HCY levels. The incidence of MetS at the 6-year follow-up was the highest in participants with concomitant high hsCRP and HCY levels, regardless of obesity. Even after adjusting for potential confounding factors including body mass index in a multivariate logistic regression model, subjects with elevated hsCRP and HCY levels had a 2.50-fold increased risk of developing MetS at the six-year follow-up compared to those who did not have high hsCRP and HCY level. MetS is more prevalent in the concurrent presence of elevated hsCRP and HCY levels. The combination of the two conditions may contribute to an increased risk of MetS, but these factors may not be synergistic.

## Introduction

Metabolic syndrome (MetS) is characterized by a cluster of predisposing factors including central obesity, insulin resistance, dyslipidemia, and hypertension (HTN), which directly increase the risk of cardiovascular diseases (CVD). These relationships have been confirmed in several longitudinal studies [1]. These components of MetS are interconnected with various risk factors such as age, genetic variations related to inflammation, and various lifestyle factors [2–7]. Among these predisposing factors, visceral obesity is the most prevalent manifestation of MetS [8]. Considering the rapidly increasing prevalence of obesity, the substantial attention to MetS-related comorbidities is expected to increase [9].

C-reactive protein (hsCRP), which is known to be an important biomarker of CVD, is an acute-phase protein that is generated in the liver and is stimulated by pro-inflammatory cytokines including TNF-α and IL-6 [10]. Substantial evidence shows that obese individuals have higher hsCRP level than non-obese individuals [11, 12], indicating that the abdominal adipose tissue may be involved in the production and regulation of hsCRP level [13–15]. A number of studies have also demonstrated that high hsCRP level is associated with increased risk for CVD and MetS [16–18], suggesting that hsCRP could be an important marker for the prediction of cardio-metabolic risk [19, 20]. Homocysteine (HCY) is a non-essential amino acid that is biosynthesized from methionine. In the last few decades, it has been suggested that an elevated HCY level is pathophysiologically involved in the promotion of cardiovascular events and increased risk of MetS [21–23]. Although previous studies reported that elevated hsCRP and HCY levels are both associated with increased risk of MetS, to the best of our knowledge, no study has examined their combined effects on the risk of MetS in a large prospective cohort study. We hypothesized that the presence of elevated HCY and hsCRP levels would accelerate the risk of MetS in a cohort study.

## Materials and methods

### Subjects

Participants in the present study were part of a larger study, the Korean Genome and Epidemiology Study (KoGES), which is an ongoing, population-based cohort study that started in 2001 under the original title “The Korean Health and Genome Study.” Detailed information on the study design and aims of the KoGES have been previously reported [24]. In brief, the original study was designed to establish a representative adult cohort in the city of Ansan, Korea, and to identify the epidemiologic characteristics, frequency, and determinants of chronic diseases among Koreans. It began in June 2001 with 5,015 participants (2,521 men and 2,494 women; ages, 40–69 years). The participants were followed biennially, with a scheduled visit for a comprehensive health examination and on-site interviews at Korea University Ansan Hospital. Data from the 4^th^ examination, conducted from March 2007 to February 2009 (for baseline assessment), and the 7^th^ examination, conducted between March 2013 and February 2015 (for the 6-year follow-up assessment), were used in the present study. All participants signed an informed consent form, and the study protocol was approved by the Institutional Review Board of Korea University Ansan Hospital (Protocol ID: ED0624). All methods and experiments were performed in accordance with the relevant guidelines and regulations. Among 3,255 participants who were initially screened at baseline, 3,170 were included in the study. All participants underwent anthropometric measurements and a general health examination. Participants were excluded if they had any known systemic inflammatory disease or genetic abnormality and extreme outliers of hsCRP (n = 56, hsCRP > 10 mg/dL) and HCY (n = 29, HCY >30 μmol/L) [25, 26]. High hsCRP was defined as >1.43 mg/dL for men and >1.20 mg/dL for women, and high HCY was defined as >14.71 μmol/L for men and >11.03 μmol/L for women, which correspond to the 75th percentiles of the whole study cohort. For the purpose of the present study, participants were divided into 4 groups based on the presence of high hsCRP and HCY level. The population was also sub-divided into tertiles of hsCRP and HCY levels for sub-group analysis.

### Anthropometric and biochemical measurements

All subjects were surveyed for age, sex, marital status, smoking, drinking, and other habits. Blood pressure (BP) was measured in a standardized manner using a mercury sphygmomanometer. Body mass index (BMI) was calculated as weight in kilograms divided by height in meters squared. Waist circumference was measured at the midpoint between the lower rib margin and the iliac crest in a standing position. Blood was drawn for biochemical analysis after overnight fasting. Biochemical data including glucose, total cholesterol, triglycerides, HDL cholesterol, and hsCRP levels were determined using an auto-analyzer (ADVIA 1650 and 1800, Siemens, Tarrytown, NY) at Seoul Clinical Laboratories (Seoul, Korea). HCY level was measured using a chemiluminescence immunoassay assay.

### Definition of MetS

Data were also collected on factors used to define components of MetS, which was defined based on the Third Report of the National Cholesterol Education Program Expert Panel III on Detection, Evaluation, and Treatment of High Blood Cholesterol in Adults (NCEP-III) [27]. The components included abdominal obesity, low HDL-cholesterol, hypertension, hypertriglyceridemia, and high fasting glucose. High blood pressure was defined when the systolic blood pressure was 130 mmHg or higher, diastolic blood pressure was 85 mmHg or higher, or the patient was taking antihypertensive medication. Low HDL-cholesterol was defined as lower than 40 mg/dL for males and 50 mg/dL for females. Hypertriglyceridemia was defined as triglycerides of 150 mg/dL or greater. High fasting glucose was defined as a glucose level of at least 100 mg/dL or when a subject was receiving oral hypoglycemic agents or insulin therapy. Abdominal obesity was defined as a waist circumference wider than 90 cm in males and 85 cm in females [28]. The metabolic score was calculated by summing the number of positive diagnostic criteria on the NCEP-III. The incidence of MetS was defined as the percentage of participants who were newly diagnosed with MetS during the 6-year follow-up period.

### Statistical analysis

Differences in the means for normal variables were examined by one-way ANOVA. The probabilistic distribution for non-normal variables was compared with the Kolmogorov-Smirnov test. Categorical variables were expressed as n (%) and analyzed with a chi-square test. Multivariate logistic regression was applied with adjustment for the factors identified to be significant risk factors for MetS in univariate analyses, which were age, sex, smoking status, alcohol use, baseline BMI, and change in BMI at the six-year follow-up (*Δ*BMI). The Bonferroni correction was applied for pairwise comparisons. Adjusted odds ratios were estimated with 95% confidence intervals (CIs), referenced to participants with normal hsCRP and HCY levels. All statistical analyses were performed using IBM SPSS version 23.0 (IBM Corp., Armonk, NY, USA). Statistical significance was identified at the 0.05 significance level.

## Results

### General characteristics of study participants

**Fig 1:**
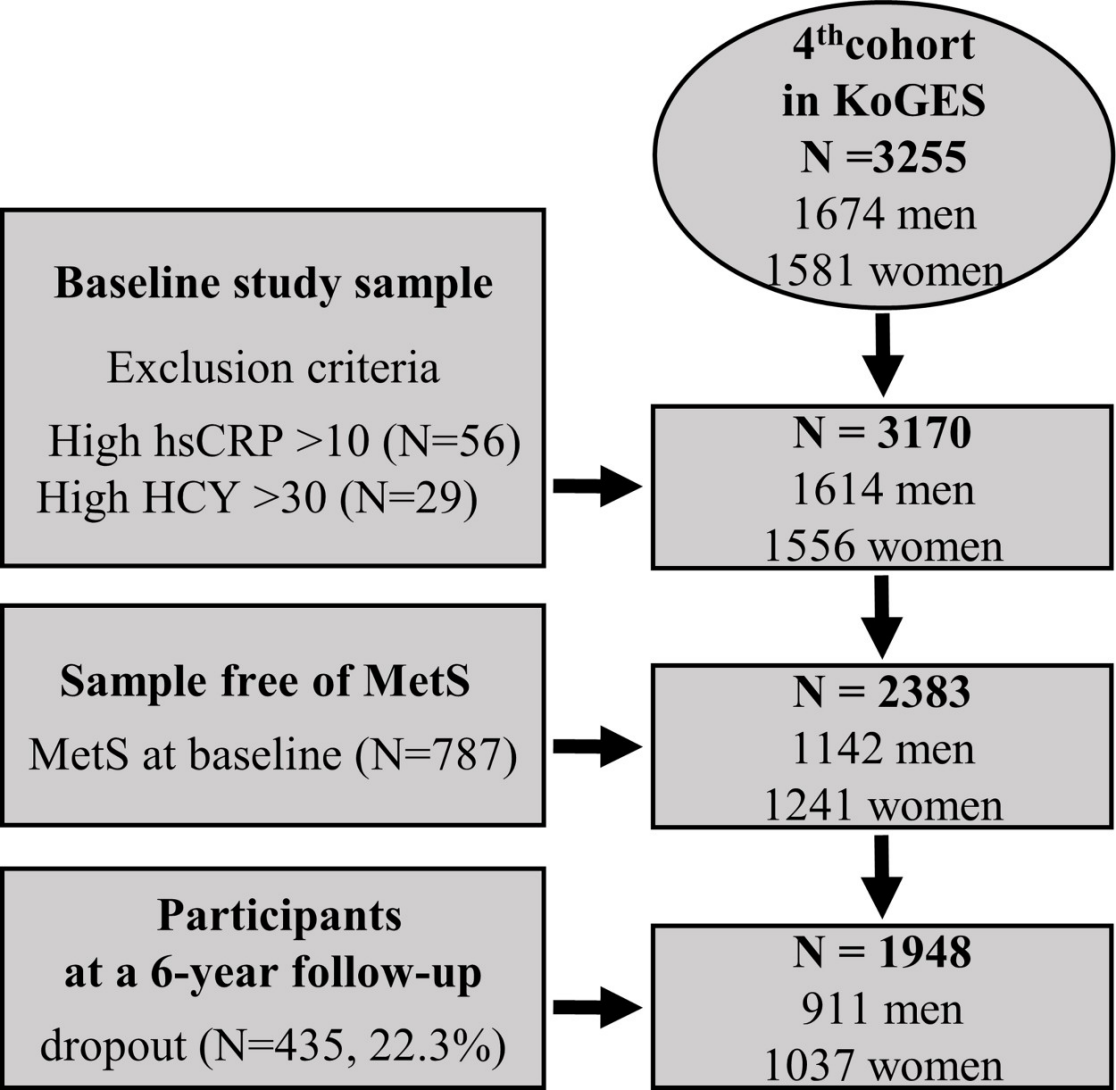
Flow chart of the selection procedure for participants in the present study.

Originally, the KoGES was designed to establish a representative adult cohort and to identify the epidemiologic characteristics and determinants of chronic diseases among Koreans in 2001. Measurement of HCY level in the KoGES participants was included in the study protocol at the 4^th^ examination. Thus, among 3,255 participants who were initially screened at baseline (4^th^ examination), 3,170 were included in the study (Fig 1). Table 1 shows the demographic and biochemical characteristics of participants at baseline, classified into four groups according to the presence or absence of high hsCRP and HCY. Among the 3170 participants at the baseline examination, 435 dropped out over the 6-year follow-up period (non-response rate, 22.3%). There were no significant differences between the respondents and the non-respondents with regard to gender, smoking status, alcohol use, and BMI (p>0.05). However, the mean age was significantly higher in non-respondents than in respondents (p<0.05). Among participants with low hsCRP, the average HCY for those in the “low” and “high” groups at baseline was 10.2 and 15.5 μM, respectively; among the participants with high hsCRP, the average HCY for those in the “low” and “high” groups was 10.4 and 15.4 μM, respectively. Systolic and diastolic BP, glucose, triglycerides, total cholesterol, and HDL cholesterol levels were significantly different among the four groups (p<0.01). Moreover, the group with high hsCRP and HCY levels had the highest metabolic score.

**Table 1:** General characteristics of participants according to the presence of high HCY and hsCRP levels.

	hsCRP[-]		hsCRP[+]		P value
	HCY[-]	HCY[+]	HCY[-]	HCY[+]	
Sample size, n (%)	1813 (57.2)	563 (17.8)	565 (17.8)	229 (7.2)	-
Age (years)	53.4±6.6	56.4±8.3§	54.8±7.2#	58.3±8.5*	<0.001
BMI (kg/m2)	24.2±2.7	24.5±2.8	25.5±3.3#	25.6±2.9*	<0.001
ΔBMI at follow-up (kg/m2)	0.14±1.21	0.03±1.28	0.20±1.15#	-0.09±1.29	0.07
Men, n (%)	911 (50.2)	298 (52.9)	300 (53.1)	105(45.9)	0.2
Current smoker, n (%)	267 (14.7)	94 (16.7)	107 (18.9)	40 (17.5)	0.11
Current drinker, n (%)	926 (51.1)	288 (51.2)	284 (50.3)	106 (46.3)	0.58
Medication for hypertension, n (%)	356 (19.6)	152 (27.0)	139 (24.6)	72 (31.4)	<0.001
Medication for diabetes, n (%)	121 (6.7)	47 (8.3)	46 (8.1)	29 (12.7)	<0.05
Systolic blood pressure (mmHg)	110.0±13.7	113.6±14.5§	112.4±14.1#	116.3±14.3*	<0.001
Diastolic blood pressure (mmHg)	73.9±9.5	75.8±10.3§	75.7±9.9#	76.3±9.7*	<0.001
Fasting glucose (mg/dL)	97.5 ±29.2	98.5±29.7	102.4±33.6#	104.6±32.3*	<0.001
Total cholesterol (mg/dL)	198.8±34.3	202.3±34.3§	203.4±36.2#	206.7±35.9*	<0.001
HDL cholesterol (mg/dL)	46.0±10.8	45.5±11.2	42.8±10.0#	41.2±8.7*	<0.001
Triglycerides (mg/dL)	130.6±78.8	143.1±95.2§	157.9±102.1#	176.6±99.3*	<0.001
hsCRP at baseline (mg/dL)	0.54±0.32	0.59±0.34d	2.86±1.8#	2.83±1.57*	<0.001
(log-transformed)	(-0.36±0.31)	(-0.32±0.30)	(0.39±0.22)	(0.40±0.21)	
hsCRP at follow-up (mg/dL)	0.83±1.11	1.02±1.22	1.55±1.57#	1.90±1.80*	<0.001
(log-transformed)	(-0.26±0.36)	(-0.16±0.37)	(0.02±0.38)	(0.11±0.38)	
HCY at baseline (μmol/L)	10.2±2.11	15.5±3.41§	10.4±2.04	15.4±3.38*	<0.001
(log-transformed)	(1.00±0.09)	(1.18±0.09)	(1.01±0.09)	(1.18±0.09)	
HCY at follow-up (μmol/L)	12.3±3.14	16.0±5.2§	12.7±3.0	16.8±5.9*	<0.001
(log-transformed)**	(1.07±0.09)	(1.18±0.12)	(1.09±0.09)	(1.20±0.12)	
Vitamin intake, n (%)	513 (28.3)	98 (17.4)	149 (26.4)	35 (15.3)	<0.001
Metabolic score	1.31±1.15	1.55±1.23§	1.88±1.23#	2.22±1.27*	<0.001
Metabolic score at follow-up	1.27±1.08	1.55±1.08§	1.61±1.18#	1.92±1.24*	<0.001

Abbreviation: BMI, body mass index; HDL, high-density lipoprotein; hsCRP, high sensitivity C-reactive protein; HCY, homocysteine.

Scale variables are summarized as mean±SD.

** Data from 2802 subjects at 6-year follow-up were included in the analysis.

^#^*P* <0.05, [HCY (-), hsCRP (+)] vs. [HCY (-), hsCRP (-)].

^§^*P* <0.05, [HCY (+), hsCRP (-)] vs. [HCY (-), hsCRP (-)].

*P <0.05, [HCY (+), hsCRP (+)] vs. [HCY (-), hsCRP (-)].

### Odd ratios for risk of MetS according to the concurrent presence of high hsCRP and Hcy levels

The prevalence and incidence of MetS among the four groups stratified by the presence of high hsCRP and HCY levels are shown in Tables 2 and 3. The prevalence of MetS was significantly different between the groups (hsCRP[–]/HCY[–] vs. hsCRP[–]/HCY[+] vs. hsCRP[+]/HCY[–] vs. hsCRP[+]/HCY[+], 17.9% vs. 27.8% vs. 35.0% vs. 47.2%, p<0.001). Moreover, there was also a significant difference in the incidence of MetS at the 6-year follow-up among the four groups (hsCRP[–]/HCY[–] vs. hsCRP[–]/HCY[+] vs. hsCRP[+]/HCY[–] vs. hsCRP[+]/HCY[+], 14.1% vs. 20.2% vs. 20.3% vs. 33.0%, 22.3 vs. 26.3 vs. 33.8 vs. 58.6 per 1,000 person-year, p<0.001). In order to examine the odds ratios for the likelihood of MetS according to the presence of high hsCRP and HCY levels, univariate and multiple logistic regression analyses were performed. The univariate analysis showed that the odds ratios for MetS in participants with either high hsCRP or HCY level were 2.45 (95% CI, 1.99–3.03; p<0.001) and 1.77 (95% CI, 1.42–2.21; p<0.001) compared to participants with low hsCRP and HCY levels, respectively. The multivariate analysis with adjustment for age, gender, smoking, alcohol status, vitamin supplement intake, and BMI showed that participants with the concurrent presence of high hsCRP and HCY levels had a 2.70-fold increase (95% CI, 1.95–3.73; p<0.01) in the risk of MetS compared to those with low hsCRP and HCY levels (Table 2). Moreover, Table 3 shows adjusted odds ratios for the estimated risk of developing MetS at the 6-year follow-up among the four groups of participants who did not have MetS at baseline (n = 2383). After adjustment for age, gender, smoking, alcohol status, vitamin supplement intake, BMI at baseline, and ΔBMI at the 6-year follow-up in a regression model, the group with high hsCRP and HCY levels had a significantly higher risk of developing MetS upon follow-up (OR, 2.50, 95% CI, 1.50–4.16; p<0.001) compared to the group with low hsCRP and HCY levels (Table 3).

**Table 2:** Estimated odds ratios for the risk of MetS according to the combination of high hsCRP and HCY levels at baseline.

	Estimated odds ratio (95% confidence interval)				P value
	hsCRP[-]		hsCRP[+]		
	HCY[-]	HCY[+]	HCY[-]	HCY[+]	
Hypertriglyceridemia, n (%)	501 (27.6)	192 (34.0)	233 (41.4)	117 (51.1)	<0.001
Unadjusted	Reference	1.36 (1.11–1.66)**§**	1.84 (1.51–2.24)*	2.74 (2.07–3.62)*	<0.001
Adjusted, Model 1	Reference	1.33 (1.08–1.64)**§**	1.82 (1.49–2.22)*	2.86 (2.14–3.83)*	<0.001
Adjusted, Model 2	Reference	1.23 (0.99–1.53)	1.40 (1.13–1.72)**§**	2.20 (1.63–2.97)*	<0.001
Low HDL cholesterol, n (%)	886 (48.9)	292 (51.7)	352 (62.5)	154 (67.2)	<0.001
Unadjusted	Reference	1.13 (0.93–1.36)	1.73 (1.43–2.10)*	2.15 (1.61–2.87)*	<0.001
Adjusted, Model 1	Reference	1.10 (0.90–1.35)	1.80 (1.46–2.18)*	2.00 (1.46–2.68)*	<0.001
Adjusted, Model 2	Reference	1.05 (0.86–1.28)	1.53 (1.24–1.88)*	1.66 (1.22–2.27)**§**	<0.001
Hypertension, n (%)	513 (28.3)	262 (46.4)	212 (37.7)	127 (55.5)	<0.001
Unadjusted	Reference	2.21 (1.82–2.68)*	1.52 (1.25–1.86)*	3.16 (2.39–4.17)*	<0.001
Adjusted, Model 1	Reference	1.90 (1.54–2.31)*	1.43 (1.16–1.75)*	2.56 (1.91–3.43)*	<0.001
Adjusted, Model 2	Reference	1.80 (1.46–2.22)*	1.09 (0.88–1.35)	2.00 (1.47–2.68)*	<0.001
High fasting glucose, n (%)	422 (23.3)	138 (24.4)	188 (33.4)	90 (39.3)	<0.001
Unadjusted	Reference	1.070 (0.86–1.34)	1.64 (1.34–2.02)*	2.13 (1.60–2.84)*	<0.001
Adjusted, Model 1	Reference	0.93 (0.74–1.18)	1.58 (1.28–1.96)*	2.00 (1.48–2.71)*	<0.001
Adjusted, Model 2	Reference	0.83 (0.66–1.06)	1.20 (0.96–1.50)	1.51 (1.10–2.06)#	<0.001
MetS, n (%)	325 (17.9)	157 (27.8)	197 (35.0)	108 (47.2)	<0.001
Unadjusted	Reference	1.77 (1.42–2.21)*	2.45 (1.99–3.03)*	4.09 (3.07–5.44)*	<0.001
Adjusted, Model 3	Reference	1.45 (1.13–1.86)**§**	1.65 (1.30–2.09)*	2.70 (1.95–3.73)*	<0.001

Abbreviation: BMI, body mass index; HDL, high-density lipoprotein; hsCRP, high sensitivity C-reactive protein; HCY, homocysteine; MetS, metabolic syndrome.

Model 1: adjusted for age, sex, current smoking, current drinking, and vitamin intake.

Model 2: adjusted for Model 1 and waist circumference.

Model 3: adjusted for Model 1 and BMI.

^#^*P* <0.05

^§^*P* <0.01

*P <0.001.

**Table 3:** Estimated odds ratio for the risk of MetS according to the combination of high hsCRP and HCY levels at 6-year follow-up.

	Estimated odds ratio (95% confidence interval)				P value
	hsCRP[-]		hsCRP[+]		
	HCY[-]	HCY[+]	HCY[-]	HCY[+]	
No. of subjects, n (%)	1228 (63.0)	317 (16.3)	306 (15.7)	97 (5.0)	-
Hypertriglyceridemia, n (%)	256 (20.8)	79 (24.9)	87 (28.4)	33 (34.0)	0.002
Unadjusted	Reference	1.26 (0.94–1.68)	1.51 (1.14–2.00)**§**	1.96 (1.26–3.05)**§**	<0.001
Adjusted, Model 1	Reference	1.32 (0.98–1.77)	1.50 (1.13–2.00)**§**	2.20 (1.40–3.47)*	<0.001
Adjusted, Model 2	Reference	1.29 (0.96–1.74)	1.39 (1.04–1.86)#	1.98 (1.25–3.13)**§**	<0.001
Low HDL cholesterol, n (%)	503 (41.0)	144 (45.4)	137 (44.8)	52 (53.6)	0.05
Unadjusted	Reference	1.20 (0.94–1.54)	1.17 (0.91–1.50)	1.67 (1.10–2.52)#	<0.05
Adjusted, Model 1	Reference	1.21 (0.93–1.56)	1.16 (0.90–1.50)	1.64 (1.07–2.51)#	<0.05
Adjusted, Model 2	Reference	1.17 (0.90–1.52)	1.04 (0.80–1.35)	1.42 (0.92–2.19)	0.19
Hypertension, n (%)	381 (31.0)	142 (44.8)	118 (38.6)	47 (48.5)	<0.001
Unadjusted	Reference	1.80 (1.40–2.32)*	1.40 (1.08–1.81)#	2.09 (1.38–3.17)*	<0.001
Adjusted, Model 1	Reference	1.54 (1.18–2.00)**§**	1.33 (1.02–1.73)#	1.63 (1.06–2.52)#	<0.01
Adjusted, Model 2	Reference	1.50 (1.15–1.95)**§**	1.20 (0.91–1.57)	1.43 (0.92–2.22)	0.02
High fasting glucose, n (%)	198 (16.1)	60 (18.9)	65 (21.2)	23 (23.7)	<0.05
Unadjusted	Reference	1.21 (0.88–1.67)	1.40 (1.03–1.92)**§**	1.62 (0.99–2.64)	<0.01
Adjusted, Model 1	Reference	1.10 (0.79–1.52)	1.33 (0.97–1.82)	1.43 (0.86–2.37)	<0.05
Adjusted, Model 2	Reference	1.05 (0.75–1.46)	1.17 (0.85–1.62)	1.20 (0.72–2.00)	0.27
Metabolic syndrome, n (%)	173 (14.1)	64 (20.2)	62 (20.3)	32 (33.0)	<0.001
Unadjusted	Reference	1.54 (1.12–2.12)**§**	1.55 (1.12–2.14)**§**	3.00 (1.91–4.72)*	<0.001
Adjusted, Model 3	Reference	1.24 (0.87–1.77)	1.20 (0.84–1.72)	2.50 (1.50–4.16)*	<0.001

Abbreviation: BMI, body mass index; HDL, high-density lipoprotein; hsCRP, high sensitivity C-reactive protein; HCY, homocysteine; MetS, metabolic syndrome.

Model 1: adjusted for age, sex, current smoking, current drinking, and vitamin intake.

Model 2: adjusted for Model 1 and waist circumference.

Model 3: adjusted for Model 1, BMI at baseline, and BMI change at follow-up (*Δ*BMI).

^#^*P* <0.05

^§^*P* <0.01

*P <0.001.

### Odds ratios for the risk of MetS according to the combination of hsCRP and HCY tertiles

**Fig 2:**
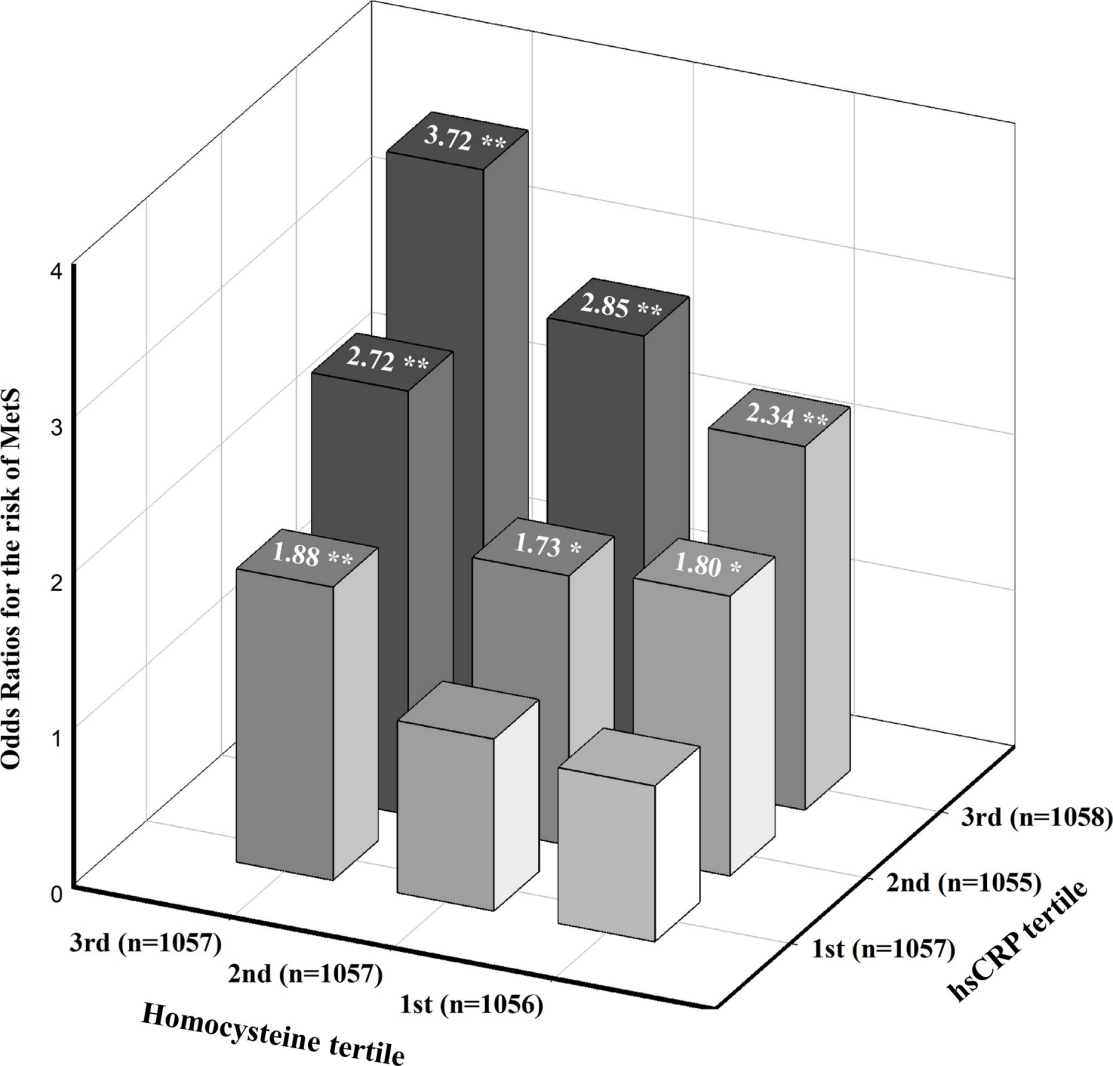
Estimated odds ratios for risk of MetS according to hsCRP and HCY tertiles. The odds ratio was estimated after adjusting for age, sex, smoking and alcohol status, vitamin intake, and BMI (n = 3170). P-value for interaction = 0.45, *P <0.05, **P <0.01.

**Fig 3:**
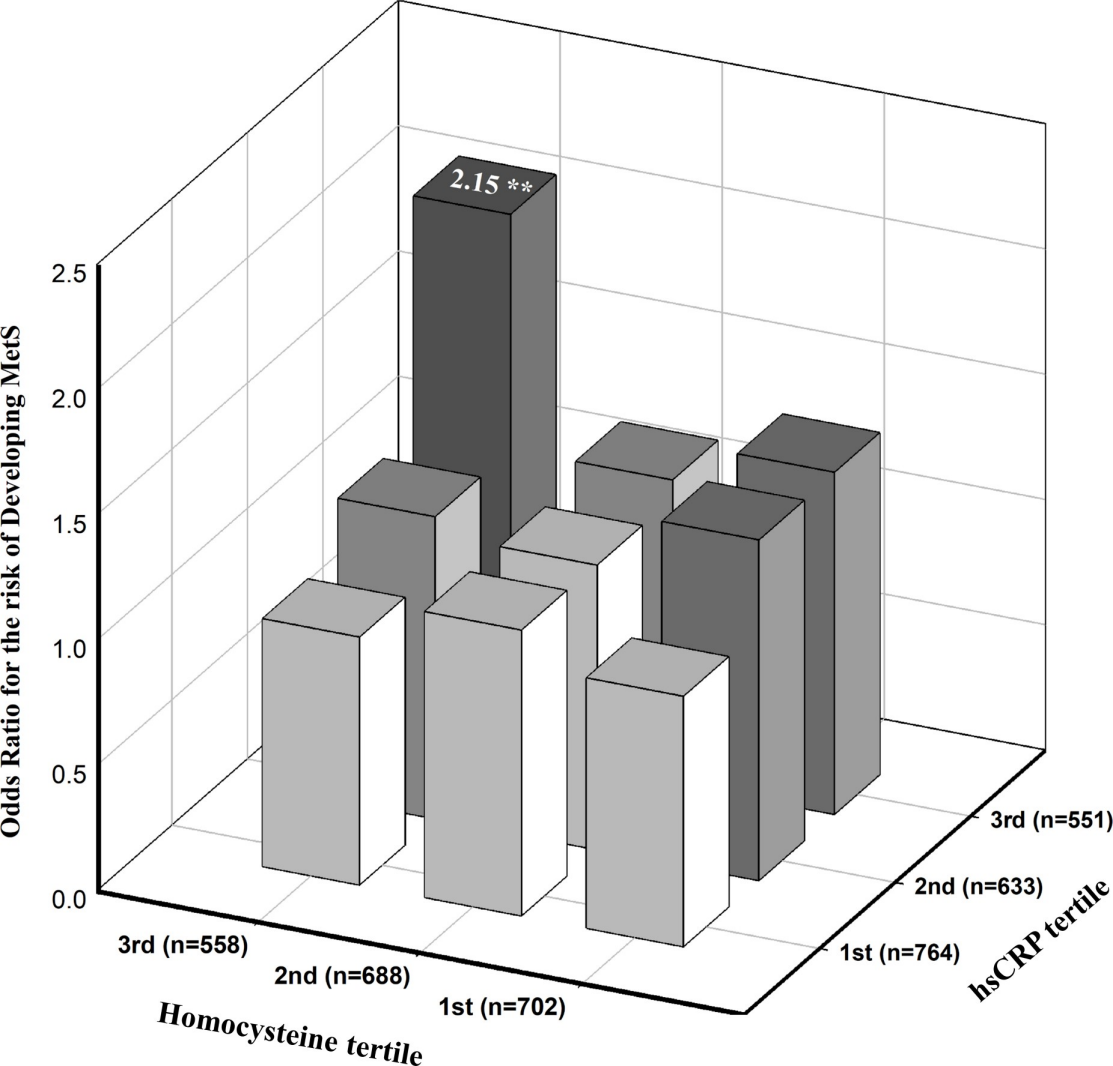
Estimated odds ratios for risk of the development of MetS according to hsCRP and HCY tertiles at a 6-year follow-up. The odds ratio was estimated after adjusting for age, sex, smoking and alcohol status, vitamin intake, BMI at baseline and change in BMI at the 6-year follow-up (n = 1948). P-value for interaction = 0.90, *P <0.05, **P <0.01.

Fig 2 presents the odds ratios for the estimated likelihood of MetS according to hsCRP and HCY tertiles. The multivariate analysis with adjustment for age, gender, smoking, alcohol status, vitamin supplement intake, and BMI showed that the odds ratios for MetS among the middle (0.42 mg/dl≤ hsCRP <1.01 mg/dl) and highest tertiles of hsCRP (hsCRP ≥1.01 mg/dl) with the highest tertile of HCY level (≥12.26 μmol/L) were 2.85 (95% CI, 1.82–4.47; p<0.01) and 3.72 (95% CI, 2.40–5.76; p<0.01), respectively, compared to those with the lowest hsCRP and HCY levels. In addition, the adjusted odds ratios for the risk of developing MetS at the 6-year follow-up among participants classified by similar cut-off points of hsCRP and HCY (Fig 3). Adjustments for age, gender, smoking, alcohol status, vitamin supplement intake, BMI at baseline, and ΔBMI at the 6-year follow-up showed that participants with the highest tertiles of hsCRP and HCY levels had a 2.15-fold increased risk of developing MetS (95% CI, 1.25–3.74; p<0.01) compared to participants with the lowest hsCRP and Hcy levels. We investigated the interactions between hsCRP and HCY tertiles and their effects on the risk of MetS after adjusting for age, gender, smoking, alcohol status, vitamin supplement intake, BMI at baseline, and ΔBMI at the 6-year follow-up. No significant interactions were observed (Figs 2 and 3, p>0.05).

## Discussion

In a large population-based cohort study, we found that MetS was prevalent among participants with the concurrent presence of high hsCRP and HCY levels. The prevalence and incidence of MetS were highest in participants with elevated hsCRP and HCY levels (Tables 2 and 3), indicating that the combination of these conditions may exacerbate the risk for MetS. Even when adjusting for potential confounding factors in logistic regression models, participants with high hsCRP and HCY levels had a 2.70- fold risk of MetS at baseline and a 2.50-fold increased risk for developing MetS at the 6-year follow-up compared to those with low hsCRP and HCY levels. Moreover, in a model that investigated the risk of MetS and hsCRP and HCY tertiles, participants with the highest hsCRP (hsCRP ≥1.01 mg/dl) and HCY tertiles (≥12.26 μmol/L) had a 3.72-fold and a 2.15-fold increased risk of MetS at the 6-year follow-up compared to those with the lowest tertile of hsCRP and Hcy levels after adjustment for potential confounding factors. However, no significant interactions between the tertiles of hsCRP and Hcy levels were examined, suggesting that the two factors may not synergistically affect the increased risk of MetS. However, to the best of our knowledge, this is the first study to investigate the combinatory effect of elevated hsCRP and HCY on the development of MetS using data from a large prospective cohort study. Therefore, the magnitude and significance of the modifying effect of high hsCRP and HCY levels in individuals on the risk of MetS should be confirmed in other cohort studies.

Considering the ever-increasing body of evidence regarding MetS, chronic low-grade inflammation, accumulation of reactive oxygen species, and activation of the immune system may have an important role in the pathogenesis of obesity-related metabolic disorders [10, 29]. Previous evidence from clinical and epidemiologic studies suggested that elevated hsCRP and HCY levels share many common pathophysiological pathways inflicting damage on the cardiovascular system [30]. However, it is unclear whether the coexistence of elevated hsCRP and HCY levels accelerates the risk of MetS. HsCRP is a robust biomarker of underlying systemic inflammation and appears to be an important marker in both cardiovascular disease and metabolic syndrome [31, 32]. While the pathophysiologic mechanisms by which systemic inflammation leads to increased risk of MetS are not clearly evident, both adipokines produced in hypertrophic adipocytes [10] and production of pro-inflammatory cytokines such as TNF-α, IL-6, and monocyte chemoattractant protein-1 released from adipose tissue of increased infiltration by macrophages may play an important role in the pathogenesis of MetS [33, 34]. In addition, data from clinical and epidemiologic studies also shows that elevated hsCRP level in healthy individuals is associated with increased risk of MetS [35] as well as future development of insulin resistance and diabetes [36] in healthy individuals. HCY is a thiol-containing intermediate amino-acid in the methionine to cysteine pathway. Over the past several decades, a number of studies reported that elevated HCY was related to endothelial dysfunction, arterial intimal-media wall thickening, and a pro-thrombotic state, providing a pathophysiologic explanation for the increased risk of CVD morbidity and mortality [21]. In animal studies, insulin affected the activity of enzymes involved in HCY turnover, and hyperhomocysteinemia has been suggested as a possible additional component of MetS [37]. Even though several population-based studies showed a significant association between MetS and plasma HCY level [38], results from these studies were limited due to cross-sectional design, small sample size, and inadequate control of confounders [39]. Therefore, a potential causal relationship between HCY level and MetS has not been clearly established. Recent evidence from a 4.8-year longitudinal study in a Chinese population also reported no significant association between baseline HCY and the incidence of MetS [40]. We found similar results; the highest prevalence and incidence of MetS at the 6-year follow-up were among participants with high hsCRP and HCY levels. However, the incidence rate of MetS among individuals with elevated hsCRP and HCY levels relative to those without them might be influenced by the high rate of non-respondents among subjects with high hsCRP and HCY levels at the 6-year follow-up, although this was not statistically different (non-respondents, n = 435; hsCRP[–]/HCY[–] vs. hsCRP[–]/HCY[+] vs. hsCRP[+]/HCY[–] vs. hsCRP[+]/HCY[+], 17.5% vs. 21.9% vs. 16.8% vs. 19.8%, p = 0.175). Moreover, elevated hsCRP level and elevated HCY level alone were significantly associated with increased risk of MetS at baseline; however, these were not predictive of the incidence of MetS at 6-year follow-up when compared to those with low hsCRP and HCY levels. Only the group with elevated hsCRP and HCY was significantly associated with increased risk of MetS, suggesting that the combination may exacerbate the risk for MetS. Therefore, the magnitude and significance of the modifying effects of elevated hsCRP and HCY on the development of MetS should be confirmed in other cohort studies.

The current study has several strengths that lend confidence to the findings. First, this study is the first to show that the co-existence of high hsCRP and HCY is correlated with development of MetS in a large general population. Second, this was a longitudinal study, which allowed us to establish a causal relationship of hsCRP and HCY levels with MetS. Since the characteristics of the study population including biochemical data, metabolic scores, BMI, hsCRP, HCY level, and blood pressure were not significantly different between participants and non-participants at baseline (p>0.05), our results are likely representative of the general population.

Although our study adapted a prospective design with follow-up measurements and used a large general population sample, several limitations should be acknowledged. First, we used hsCRP as a marker to define the inflammatory status of the participants, but the use of only hsCRP may not sufficiently reflect the inflammatory status of participants. The combined effects of other inflammatory cytokines and HCY on the development of MetS should be examined in further studies. Second, the group with high hsCRP and HCY levels has a relatively small sample size compared to the other groups. Third, mild to moderate elevation of HCY level in the general population may be partially influenced by participant nutritional habits and exercise status; these factors were not considered in the present study. Our data showed that participants with regular intake of any type of vitamin supplement had significantly lower levels of HCY compared to those who did not take vitamin supplements. Even though the effect of vitamin supplementation on reducing the risk of type 2 diabetes and CVD is still controversial [41, 42], several lines of research suggest that vitamin supplementation with folate, B6, and B12 has the potential to lower HCY level and thus cardio-metabolic risk [43]. Finally, genetic variation or epigenetic modifications that might play an important role in the elevation of hsCRP and HCY level were not examined in the present study. Accordingly, future longitudinal studies that focus on interactions between hsCRP- and HCY-related genetic variations and the development of MetS should be conducted.

In conclusion, MetS is more prevalent in the concurrent presence of elevated hsCRP and HCY levels. Taken together, combined elevation of hsCRP and HCY levels were associated with increased risk of MetS, but these factors may not be synergistic. Additional longitudinal study is needed to further address the significance and magnitude of the coexistence of these conditions on the development of MetS in the context of reducing CVD risk.
